# The Effect of Urbanization on Health Care Expenditure: Evidence From China

**DOI:** 10.3389/fpubh.2022.850872

**Published:** 2022-02-15

**Authors:** Qi Shao, Ran Tao, Magda Mihaela Luca

**Affiliations:** ^1^China Center for Human Capital and Labor Market Research, Central University of Finance and Economics, Beijing, China; ^2^Qingdao Municipal Center for Disease Control & Prevention, Qingdao, China; ^3^Department of Dentistry, Victor Babeş University of Medicine and Pharmacy, Timisoara, Romania

**Keywords:** urbanization, health care expenditure, population aging, panel threshold regression model, China

## Abstract

This paper investigates the impact and non-linear effects of urbanization on health care expenditure in China. The results indicate that urbanization in both Eastern and Central regions can significantly increase health care expenditure. But the impact of urbanization is not significant, which is related to the backward economic development level and low urbanization rate in the Western region. Taking population aging into consideration, the results of the panel threshold regression model imply that the positive relationship between urbanization and health care expenditure becomes greater when the level of population aging exceeds 10.72% in the Eastern region and 7.00% in the Western region. Therefore, in the urbanization process, the government should pay attention to the positive effect of urbanization on health care expenditure, provide more financial support for the construction of medical facilities, and expand the coverage of medical services and security for residents, especially for elderly people.

## Introduction

With the development of industrialization, urbanization is expanding all over the world (1). Urbanization is an inevitable trend and essential requirement to advance the progress of human society (2, 3), which boosts economic growth by expanding demand (4). According to The United Nations, in 2020, more than 55% of the world's population live in cities and it is predicted that this proportion will reach 70% by 2050, with 96% of urban growth occurring in developing countries (5). Urbanization means the whole process of qualitative change from a rural lifestyle to an urban lifestyle (6), which is usually considered has important consequences for people's living standards and health (7, 8). However, the impact of urbanization on the health of residents is ambiguous (9, 10). On the one hand, the progress of urbanization enables people to have more access to health services and provides better healthcare resources to them, which can improve the residents' health status (11, 12). On the other hand, urbanization is also related to sedentary, stressful lifestyles (13), unbalanced nutrition (14), and other environmental factors, such as air pollution (15), which harms people's health (10). Therefore, there are two possible opposite impacts of urbanization on the health status, so the impact on the expenditure of health care is also uncertain. As an indispensable part of residents' daily life expenditure, health care expenditure consists of the spending on medicines, as well as medical and health services, which is one of the important indicators to measure the quality of life of residents (16). If the medical burden is too heavy, it will crowd out residents' consumption in other aspects, thus even restricting the economic development of a country. Considering the two potential opposite effects, the influence from urbanization on health care expenditure deserves further in-depth discussion.

As the world's largest developing country, China's urbanization has developed rapidly since economic reform in the late 1970s (17, 18), which has been a notable global event (19). Many factors, such as internal immigration policy and surplus agricultural labor, have accelerated the speed of urbanization (20). According to The Seventh National Census, the urbanization level in China measured by population ratio is about 63.89%, which is an increase of about 46% over 1978. It is considered that the path of urbanization in China is unique because it is not the same as that of developed economies and does not repeat the model of developing countries (21, 22). Compared with other countries, China's urbanization is larger in scale, faster in speed (23), and has received strong support from the Chinese government. To guide the development of urbanization more efficiently, the Chinese government released the first official plan “National New-type urbanization Plan (2014–2020)” in 2014 (19) and “Strategic Plan for Rural Vitalization (2018–2022)” in 2017. With strong support from the government (24), the rate of urbanization in China is expected to still maintain an upward trend. It should be noted that in the process of urbanization in recent years, residents' health care expenditure is also significantly rising in China. According to the latest “Urban-rural Integration Household Income and Living Conditions Survey” of China, the per capita consumption expenditure of residents in 2020 was 21,210 yuan, an increase of 75.79% compared with 2013. The Chinese government attaches great importance to the medical care burden of residents and alleviates the burden of residents through various medical reform policies, such as the New Rural Cooperative Medical Insurance Scheme (NRCMS) and the Urban Residents Basic Medical Insurance (URBMI) (25). Considering that urbanization has brought significant changes into residents' life, it is necessary to understand the relationship between urbanization and residents' health care expenditure, which is of great practical significance for urban management and policy development in China. In addition, with the increasingly prominent aging phenomenon, the improvement of average life expectancy also leads to the continuous increase of residents' healthcare expenditure (26). In the context of a large aging population in China, whether the level of aging takes part in the influence of urbanization on medical expenditure should also be investigated.

Compared with the existing research, we have the following contributions. Firstly, existing research about the relationship between urbanization and health care expenditure is inconclusive. This paper reexamines the impact of urbanization on health care expenditure and discusses the impact among different regions in China. Secondly, based on the differences between the East, Central and Western regions, the results of this study show that urbanization can significantly increase residents' health care expenditure in the whole country as well as Eastern and Central regions. In Eastern and Western regions, the positive impact of urbanization on health care expenditure becomes greater when the population aging exceeds the threshold value, which is related to more medical services required by the elderly population. Third, no matter whether the relationship is positive or negative, previous studies have assumed that the correlation between urbanization and health care expenditure is linear. But the link among variables might be influenced by external factors and present non-linear characteristics, so the conclusions from prior investigations maybe not be convincing (27). Considering that the panel threshold regression model is suitable for scrutinizing the asymmetric relationship between variables without the requirement of non-linear equation (28), this paper uses the panel threshold regression model to study whether urbanization has an asymmetric effect on health care expenditure. Finally, some useful policy implications are provided. The government should increase financial support for the construction of medical facilities and expand the coverage of medical services for residents to gradually meet the residents' growing medical and healthcare consumption needs in the process of urbanization.

The overall structure of the study consist of seven parts. The first part is the introduction. The second part sums up the previous literature comprehensively. The third part introduces the health care expenditure model with urbanization. The fourth part presents the methodology used in this paper. The fifth part is data descriptions and the sixth part reports the results and discussions. The last part proposes the conclusion and policy implications of this study.

## Literature Review

The impact of urbanization on human beings has two sides. Urbanization can provide more access to health services, better water quality, and sanitation infrastructure. However, urban environments can also lead to stressful lifestyles, nutritionally unbalanced diets, higher metabolic and cancer risks which relate to poor health (29). The prior research about the impact of urbanization was mainly conducted in developed countries. Some hold the view that urbanization adversely affects people's health and increases health care expenditure. Rygaard (30) stated that in European in-country migration from rural to urban, the risk of family system disorganization, poor child attachment, and child abandonment also increased, which made children face a growing mental health challenge. Using data from 20 adult population surveys conducted in developed countries since 1985, Peen et al. (31) found that the prevalence of mental illness (38% higher for depression and 21% higher for anxiety) was higher in urban than in rural. Emerging evidence indicated that urbanization can affect health through environmental, economic, and social factors. For example, a large number of carbon dioxide emissions exacerbate the greenhouse effect and air pollution such as PM2.5 becomes more serious with the development of urbanization, which does harm to human health (32, 33). However, the fact that urbanization is associated with an elegant working environment, more access to medical resources, and closer social ties reflected that urbanization can have a positive effect on health (34). A study performed in Korea showed that the standardized survival rate of out-of-hospital cardiac arrest assessed by emergency medical services in metropolitan and city communities increased, but no increase in rural areas (35). Using public administrative databases of end-of-life cancer care for adult patients in Ontario, Canada, Liu et al. (36) proposed that residents in rural and remote areas struggled with worse health and more medical expenditure than residents in urban because of the restrictions of space and non-space medical accessibility. In addition, Phillips (37) held the view that no discipline or method can clearly explain the relationship between urbanization and health or the diversity of urban health problems. Because the urban environment is various, each individual has different periods and ways to expose to the urban environment. Assah et al. (38) revealed that compared with rural residents, urban dwellers in Sub-Saharan Africa had a significantly lower physical activity energy expenditure and higher prevalence of metabolic syndrome. It is claimed that being born or living in a town during childhood increases the risk of developing mental disorders in the future, especially in developing countries in which the urban population has increased violently (13). Mutatkar (39) stated that, in the metropolitan cities in India, non-communicable diseases such as diabetes, cardiovascular disease, and mental illness have increased significantly. Moreover, a large study conducted by the India national mental health survey confirmed that stress-related disorders in city areas are 2–3 times that of rural and semi-rural areas (40). Tajudeen et al. (41) employed a structural time series model (STSM) to re-examine the contributions of the key underlying drivers of public health expenditure in Tanzania and found that urbanization is an important driver of public health expenditure.

Many scholars have studied the relationship between urbanization, health, as well as health care expenditure in China. Compared with other countries, the results obtained in China by different methods in different periods were different even completely opposite, and most of the research was conducted in a certain region or city in China. Some research confirmed that although urbanization in China poses challenges to the health of residents, people who move to urban areas tend to have better health conditions than those who live in rural areas (42, 43). Chen et al. (44) found that, compared with urban children, rural children reported more anxiety and depression symptoms and worse self-reported mental health due to the lower education level and poor use of medical facilities in rural areas. Lee et al. (45) concluded that a less urbanized lifestyle has a significant association with an impairment through interviews with older adults in Taipei. Nevertheless, most studies argued that urbanization's adverse effects on people's health outweigh beneficial effects, and has brought about an increase in medical and health care expenditure. Yu et al. (46) found that urbanicity was a risk factor for hyperuricemia (HUA) in Chinese adults. Miao and Wu (12) indicated that although the income increases with the development of urbanization in China, a more high-fat diet and reduction of exercise in the process of urbanization offset the health benefits from high income. With the development of urbanization in China, the healthcare demands of people have been increasing. Lin et al. (47) indicated that in Taiwan, with the democratization of politics and the popularization of human rights thought, more people regard access to health care services as a basic human right and demand health care services. Therefore, in areas with a high degree of urbanization, it will lead to higher medical expenditure, resulting in the difference of medical resources between urban and rural areas. Using the public statistics provided by Shanghai Municipal Government, Luo et al. (48) found that the degree of urbanization has a positive effect on the personal medical expenditure due to the unfair medical resource allocation in the process of urbanization. Ahmad et al. (49) analyzed the dynamic interactive causal links between urban agglomeration, and health expenditures across developmental disparities in China through a health expenditures-augmented growth model, they found that there is a bidirectional positive causal linkage between urban agglomeration and health expenditures growth.

Although much relevant literature has explored the relationship between urbanization and health care expenditure, there is still room for further research. First, whether urbanization has a positive or negative impact on health care expenditure, existing research assumes that the impact is linear. This assumption ignores the time-varying characteristics of time series and external structural mutations, which may lead to inaccurate results. With panel threshold regression model, we can draw more accurate conclusions and fill in the gaps in this field. Secondly, the research on urbanization and healthcare expenditure in China is mainly carried out in a certain city or province, lacking comprehensive analysis from the macro level. Especially for China, a large developing country with a large population, to grasp the impact of urbanization from the macro-level is beneficial for policymakers to make overall decisions on national urbanization strategies. Finally, we examine whether urbanization has a different impact on healthcare expenditure across different regions of China.

## The Health Care Expenditure Model With Urbanization

The influence of urbanization on health care expenditure derives from the theoretical framework of Akpalu and Normanyo (50) and Jiang et al. (51). The utility function of a rational consumer is assumed to be decided by *h*, the health condition, and *c*, the consumption of the bundles of goods. The utility function can be defined as:


(1) $$\begin{array}{l} \text{u=u(c,h)} \end{array}$$

which is strictly concave in *c* and *h* and strictly increasing. And the consumer's goal is to maximize his utility. We assume that health condition is decided by health input (or health care expenditure), *I*, which can be viewed as derived demand. Influenced by a variety of exogenous factors, the marginal rate of return of *I* is partly random and partly deterministic. Uncertain factors are related to misdiagnosis and unknowingly living with various polluted harmful substances, such as industrial exhaust gas and heavy metal polluted water. Thus, the health condition of an individual can be defined as:


(2) $$\begin{array}{l} h_{t+1}=h_{t}+eI_{t} \end{array}$$

where *e* is the return to the input of health, and *h*_0_ is the initial health status. In addition, *e*=*d*-*r*, in which *d* represents the deterministic return to *I* and *r* denotes the random part.

Referring to Jiang et al. (51), the health of an individual in the next period depends on the health of the current period, the health depreciation rate, and many other influencing factors, which can also be described as:


(3) $$\begin{array}{l} h_{t+1}=\left(1-\delta_{t}\right)h_{t}+M_{t} \end{array}$$

Urbanization can provide increased access to health services, better water quality, and sanitation infrastructure, and thus improve health conditions to some extent. However, urban environments can also lead to stressful lifestyles and nutritionally unbalanced diets which are harmful to health. So, we need to take urbanization as one of the health depreciation factors. It is assumed that the health depreciation rate is in the form of Cropper:


(4) $$\begin{array}{l} \delta_{\text{t}}=\delta_{0}U_{t}^{\alpha }L_{t}^{\beta } \end{array}$$

where 0 is the health depreciation rate in the initial period, *U*_*t*_ is the degree of urbanization in period *t*, and *L*_*t*_ is the lifestyle of an individual.

The function of other influencing factors can be defined in detail in the form of Cochrane Glass (C-D) with a constant size as follows:


(5) $$\begin{array}{l} M_{t}=U_{t}^{\alpha }H_{t}^{\phi }G_{t}^{\gamma }T_{t}^{\eta }N_{t}^{\lambda } \end{array}$$

where *U*_*t*_ is the level of urbanization. *H*_*t*_ represents the foods the consumer can buy which is beneficial for health. *G*_*t*_ indicates the financial expenditure and services provided by the government in the aspect of health and medical care. *T*_*t*_ is the time required to affect health. *N*_*t*_ means other factors that can influence health such as one's age, education, income, etc.

Therefore, the health determination formula can be rewritten as:


(6) $$\begin{array}{l} h_{t+1}=\left(1-\delta_{0}U_{t}^{\alpha }L_{t}^{\beta }\right)h_{t}+U_{t}^{\alpha }H_{t}^{\phi }G_{t}^{\gamma }T_{t}^{\eta }N_{t}^{\lambda } \end{array}$$

Combining Equations (2) and (6), we can get:


(7) $$\begin{array}{l} h_{t}+eI_{t}=\left(1-\delta_{0}U_{t}^{\alpha }L_{t}^{\beta }\right)h_{t}+U_{t}^{\alpha }H_{t}^{\phi }G_{t}^{\gamma }T_{t}^{\eta }N_{t}^{\lambda } \end{array}$$

Rearranging Equation (7), we obtain:


(8) $$\begin{array}{l} I_{t}=\frac{\left(-\delta_{0}U_{t}^{\alpha }L_{t}^{\beta }h_{t}+U_{t}^{\alpha }H_{t}^{\phi }G_{t}^{\gamma }T_{t}^{\eta }N_{t}^{\lambda }\right)}{e} \end{array}$$

Then, taking the partial derivative of both sides concerning the level of urbanization, we can get:


(9) $$\begin{array}{l} \frac{\partial I_{t}}{\partial U_{t}}=\frac{\alpha U_{t}^{\alpha -1}\left(-\delta_{0}L_{t}^{\beta }h_{t}+H_{t}^{\phi }G_{t}^{\gamma }T_{t}^{\eta }N_{t}^{\lambda }\right)}{e} \end{array}$$

It is considered that the government expenditure (*G*_*t*_) and the lifestyle of an individual (*L*_*t*_) remain unchanged in the short run. The rate of influence from urbanization on one's medical care expenditure depends on the consumption of health goods (*H*_*t*_), the time needed (*T*_*t*_), and other factors (*N*_*t*_).

## Methodology

### Panel Threshold Regression Model

Due to the possible non-linear relationship between the level of urbanization and health care expenditure, this paper draws on the panel threshold model proposed by Hansen (52). The model uses the observation value of the threshold variable to estimate the appropriate threshold value, thus avoiding the insufficiency of subjective judgment partition and deriving more accurate results. Threshold models include the single threshold model, double threshold model, and multiple threshold model. We first assume that there is a single threshold, and the model is as follows (53–55):


(10) $$\begin{array}{l} \ln\;\exp_{\text{it}}=\{\begin{array}{ccc} \mu_{i}+\theta^{\prime }x_{it}\text{+}\alpha_{1}urban_{it}+\varepsilon_{it} & if & aging\leq \gamma \\ \mu_{i}+\theta^{\prime }x_{it}\text{+}\alpha_{2}urban_{it}+\varepsilon_{it} & if & aging>\gamma \end{array} \end{array}$$

where *lnexp*_*it*_ represents health care expenditure, and *urban*_*it*_, the threshold variable, is the urbanization level of province i at time t. α_1_ and α_2_ are the associated coefficients of the threshold variable. *X*_*it*_ denotes control variables and θ are the associated coefficients. γ is the estimated threshold value of population aging. μ_*i*_ is the fixed effect that varies with provinces. ε_*it*_ is the error term following normal distribution: ε_it_ ~ N(0, σ^2^).

This model can also be described as follows:


(11) $$\begin{array}{l} \ln\;\exp_{it}=\mu_{i}+\theta^{\prime }x_{it}\text{+}\alpha_{1}urban_{it}I\left(aging\leq \gamma \right)\\ \;+\alpha_{2}urban_{it}I\left(aging>\gamma \right)+\varepsilon_{it} \end{array}$$

*I(*·*)* is indicator function,and if what is in parentheses holds, *I(*·*)* = 1; otherwise, *I(*·*)* = 0.

The purpose of this study is to use the known data *lnexp*_*it*_ and *urban*_*it*_ to estimate the unknown parameters γ, α, θ and σ^2^. Equations (10) and (11) are for a single threshold, but there may be double thresholds empirically. In this case, the model can be modified to (12) or (13):


(12) $$\begin{array}{lll} \ln\;\exp_{\text{it}} & \text{=} & \{\begin{array}{ccc} \mu_{i}+\theta^{\prime }x_{it}\text{+}\alpha_{1}urban_{it}+\varepsilon_{it} & if & aging\leq \gamma_{1}\\ \mu_{i}+\theta^{\prime }x_{it}\text{+}\alpha_{2}urban_{it}+\varepsilon_{it} & if & \gamma_{1}<aging\leq \gamma_{2}\\ \mu_{i}+\theta^{\prime }x_{it}\text{+}\alpha_{3}urban_{it}+\varepsilon_{it} & if & \gamma_{2}<aging \end{array} \end{array}$$

(13) $$\begin{array}{l} \ln\;\exp_{it}=\mu_{i}+\theta^{\prime }x_{it}\text{+}\alpha_{1}urban_{it}I\left(aging\leq \gamma_{1}\right)\\ \;+\alpha_{2}urban_{it}I\left(\gamma_{1}<aging\leq \gamma_{2}\right)\\ \;+\alpha_{3}urban_{it}I\left(\gamma_{2}<aging\right)+\varepsilon_{it} \end{array}$$

Deduced by analogy, the model can be extended to models with multiple thresholds. The form of the threshold model is determined by the number of thresholds. Compared with traditional methods of non-linearity which are less fitted and lack to capture the sharp turning points, the threshold regression model can notice turning points well. The endogenous sample data can completely determine the threshold and its estimated parameters without the specific form of the non-linear regression equation (56–58).

## Data

Considering that “urbanization” has been put forward as a national strategy in China since the 10th 5-year Plan in 2001, this paper chooses the annual data of 31 provinces in China from 2001 to 2019 including a total of 589 entries. The data is collected from the China Statistical Yearbook, China Health Statistical Yearbook, and China's National Bureau of statistics.

Based on the previous research, the per capita health care expenditure (*lnexp*) is considered as the explained variable (59), which mainly consists of the cost of medicines, medical equipment, and services for medical and health care. *exp* is logarithmically processed to ensure the stability of data. The level of urbanization (*urban*) is the explanatory variable. Urbanization is accompanied by population migration from rural to urban, and rural areas gradually evolve into urban areas. The most commonly proxy for urbanization in the literature is the proportion of the urban population in the total population (53, 60). Therefore, the ratio of the urban population is used to present the urbanization level. In addition, the impact of aging on health care expenditure can not be ignored. The deepening of population aging may be accompanied by increasing demand for medical services. With the development of urbanization, medical insurance and social security funds may be increasingly transferred to retirees (61), so that the problem of “high cost of medical treatment” of residents is difficult to be further alleviated. The population aging (*aging*) is considered as the threshold variable in this paper, in which the population aging is measured by the proportion of the population aged 65 and over in the total population (62).

Five control variables are considered in this study: GDP (*lngdp*), income (*lnincome*), education level (*edu*), participation in medical insurance (*med*), and the number of medical and health institutions (*inst*). Firstly, economic development can determine residents' health care expenditure to some extent (63). Therefore, this paper takes the GDP of each province as a control variable to measure the economic development in each province. Secondly, according to Grossman's theory of health care demand, income is an important factor affecting residents' health demand (64), which can influence health care expenditures in two ways. On the one hand, the growth of income will increase the monetary value of healthy time; on the other hand, an increase in wages will bring a higher marginal cost of producing health. Therefore, people tend to purchase more health services when income increases to reduce the time spent on health investments. Since the main source of income can be regarded as wages, this paper calculates the per capita income by weighting urban and rural average wages with the proportion of the urban and rural population as the weight. Third, the improvement of education level will also affect health care expenditure. Education can improve the output efficiency of health investment, that is, the marginal productivity of both medical services and time increases. As a result, education level can improve the efficiency of residents' health investment and reduce residents' demand for medical services, thus affecting residents' medical expenditure (63). As a measure of education level, the years to complete every degree of education are weighted by the proportion of the population. Fourth, the supply of medical resources will also affect the residents' medical care expenditure. Participation in medical insurance can reduce residents' expenditure on medical care (65). This paper considers the number of employees participating in basic medical insurance to measure their participation in medical insurance. Finally, the quantity of medical and health institutions also reflects health care resources in a region (66) and is taken as one of the control variables.

To eliminate the impact of inflation, all price-related variables have been adjusted with 2001 as the base year. The statistical description of each variable is shown in Table 1. Following the classification standards of China's National Bureau of Statistics, 31 provinces are divided into Eastern, Central, and Western regions, including 11, 8, and 12 provinces, respectively^1^. Mean, median, standard deviation, skewness, kurtosis, and Jarque-Bera statistics of these three sub-samples are also shown respectively in Table 1. It is clear that, due to the developed economic level and rich medical resources, health care expenditure in the Eastern region is higher than that in the Central and Western regions. And the level of urbanization and aging in the Eastern region is also higher. In terms of the number of medical institutions, the Central region is the largest, which can be attributed to advanced facilities in some Center provinces. As for the level of income and education, the average in the Eastern region is still the largest.

**Table 1:** Descriptive statistics of the variables.

		**Mean**	**Median**	**Std. Dev**.	**Skewness**	**Kurtosis**	**Jarque-Bera**
All	lnexp	6.169	6.224	0.712	−0.265	2.517	12.63***
	urban	50.617	49.680	15.526	0.477	3.174	23.06***
	aging	9.343	9.003	2.228	0.571	3.104	32.24***
	lngdp	8.054	8.193	1.004	−0.939	3.830	103.40***
	med	15.949	8.770	20.607	2.470	9.307	1575.00***
	lnincome	9.654	9.710	0.726	−0.025	2.377	9.59***
	inst	2.221	1.574	2.012	1.494	4.686	288.90***
	edu	8.491	8.498	1.251	−0.387	5.638	185.50***
East	lnexp	6.478	6.536	0.615	−0.206	2.562	3.15
	urban	63.244	61.800	14.911	0.125	2.275	5.12*
	aging	10.222	9.997	2.144	0.701	3.023	17.10***
	lngdp	8.660	8.771	0.839	−1.190	3.903	56.46***
	med	21.850	12.073	25.317	1.922	5.895	201.70***
	lnincome	10.026	10.077	0.706	−0.122	2.435	3.30
	inst	2.300	1.527	2.262	1.408	4.065	78.95***
	edu	9.215	9.056	1.148	0.825	3.375	24.93***
Center	lnexp	6.110	6.099	0.654	−0.101	2.155	4.78*
	urban	47.117	48.275	8.853	−0.502	2.324	9.27***
	aging	9.362	8.985	1.756	0.476	2.597	6.77**
	lngdp	8.307	8.240	0.386	0.128	2.268	3.81
	med	16.979	13.318	19.114	2.595	10.637	539.90***
	lnincome	9.475	9.571	0.633	−0.252	1.860	9.84***
	inst	2.708	2.083	1.944	1.025	3.040	26.62***
	edu	8.621	8.704	0.640	−0.422	2.623	5.415*
West	lnexp	5.924	5.899	0.730	−0.204	2.285	6.43**
	urban	41.376	41.900	11.305	−0.131	2.498	3.04
	aging	8.524	8.191	2.282	0.827	3.387	27.40***
	lngdp	7.329	7.643	0.980	−0.872	2.803	29.27***
	med	9.852	5.521	14.128	2.826	12.456	1153.00***
	lnincome	9.434	9.469	0.666	−0.158	1.857	13.36***
	inst	1.824	1.194	1.722	2.034	7.812	377.10***
	edu	7.740	7.975	1.232	−1.215	4.597	80.34***

***, **, and * indicate significance levels at 1, 5, and 10%, respectively.

## Empirical Results

To avoid spurious regression and ensure the validity of the estimated results, it is of vital importance for panel data to test whether the data is stable (67). To test whether unit-roots exist in the variables used in this paper, we choose Levin-Lin-Chu (LLC) test. According to the results of the test, the *p*-value of all variables is <0.01, which means that the explanatory variable, the explained variable, and all control variables are stable. Next, the *p*-value of the Hausman test is 0.0000, indicating that the fixed effect model should be applied instead of the random effect model. Therefore, we first use the fixed effect model to identify the influence of urbanization on health care expenditure, and Table 2 displays the results.

**Table 2:** The estimated coefficients of the fixed effect model.

	**Variables**	**Estimated value**	***p*-value**	**OLS se**	**t_**OLS**_**
All provinces	urban	0.0065***	0.0100	0.0025	2.5878
	lngdp	−0.0482	0.5320	0.0772	−0.6246
	med	−0.0022***	0.0000	0.0004	−5.2021
	lnincome	0.7577***	0.0000	0.0725	10.4573
	inst	0.0196***	0.0000	0.0054	3.6260
	edu	−0.0963***	0.0000	0.0214	−4.4954
East	urban	0.0077**	0.0460	0.0038	2.0124
	lngdp	−0.2620	0.1600	0.1858	−1.4105
	med	−0.0031***	0.0000	0.0006	−4.8180
	lnincome	0.5891***	0.0000	0.1414	4.1664
	inst	0.0309***	0.0000	0.0083	3.7507
	edu	−0.0773*	0.0630	0.0413	−1.8736
Center	urban	0.0126**	0.0400	0.0061	2.0719
	lngdp	−0.0938	0.4840	0.1337	−0.7018
	med	0.0001	0.9450	0.0008	0.0686
	lnincome	0.1842	0.3810	0.2092	0.8801
	inst	−0.0079	0.4870	0.0114	−0.6969
	edu	0.0105	0.8110	0.0436	0.2399
West	urban	0.0050	0.3170	0.0050	1.0040
	lngdp	0.0083	0.9390	0.1073	0.0772
	med	−0.0004	0.6450	0.0009	−0.4613
	lnincome	0.4919***	0.0000	0.1142	4.3052
	inst	0.0289**	0.0110	0.0113	2.5561
	edu	−0.0836***	0.0080	0.0313	−2.6725

***, ** and * indicate significance levels at 1, 5, and 10%, respectively.

As can be observed from Table 2, urbanization has a significant positive impact on healthcare expenditures. The estimated coefficient of urbanization in the Eastern region and Central region is 0.0077 and 0.0126, respectively, and both are significant at a 5% significance level, while the urbanization in the Western region does not have a significant impact on healthcare expenditure.

The following explanation may be account for this result. On the one hand, with a more developed tertiary industry and eastern marine economic circle (68), the Eastern region has more employment opportunities and is much more attractive to the rural floating population, so the population flow from rural areas to urban mostly concentrated in this region. However, medical insurance does not cover the majority of the newly enrolled urban population, who are still enrolled in the new rural cooperative medical insurance, so that they are not subsidized by public medical expenses when purchasing medical services (69). New residents who purchase the same medical service have to pay more money out of their pocket, thus increasing the level of per capita medical expenditure. On the other hand, as an important part of the process of urbanization, the development of non-agricultural industries and the spatial agglomeration brings scientific and technological progress and new medical services, thus creating new demand and increasing medical care expenditure of residents. Under the combined effect of these two aspects, the construction of urbanization increases the medical and health expenditure of urban residents. As an important regional economic growth sector in China, the Central region receives the transfer of coastal industries and accelerates the development of economy, but it has also been accompanied by air pollution problems such as sulfur dioxide, soot, and PM2.5. Especially after the implementation of “Strategy of the Rising of Central region” in 2004, the trend of various pollution-intensive industries moving to the Central region has become more obvious. Many studies have found that air pollution is one of the important factors leading to the increase of health care expenditures of residents (70, 71). As for the Western region of China, due to the constraints of its low economic level, large population, vast territory, and other factors, the urbanization of the Western regions has been at a low level. Although the implementation of the western development strategy has accelerated its economic development, played a vital role in the transfer of surplus labor, and accelerated the process of urbanization to a certain extent, there still exists a large gap between the urbanization level in the Western and the Eastern regions. The Western region has always been a relatively backward urbanization area, so its impact on residents' health care expenditure is not significant.

In 2000, with the proportion of the population aged 65 and above reaching 7.0%, China began to step into an aging society. In 2019, the proportion of the population aged 65 and above reached 12.6%, indicating a deepening aging of the population. Considering the background of the increasingly serious aging in China, this paper uses PTRM to do further research.

In Table 3, the *p*-value of the single threshold effect test is 0.0806, which indicates that a single threshold effect exists in urbanization and health care expenditure with a population aging rate of 10.72% in the Eastern region. The *p*-value of the double threshold effect test is 0.1278, which confirms that there is only one threshold between the two variables. According to Table 4, in the Eastern region, when the aging level is lower than 10.72%, the coefficient of urban is 0.0034 and significant at 1% level. The development of urbanization has a significantly positive effect on health care expenditure. When the population aging exceeds 10.72%, the coefficient of urbanization on healthcare expenditure increases from 0.0034 to 0.0051. Therefore, the model shows an asymmetric non-linear relationship between urbanization level and health care expenditure. The positive relationship between urbanization and health care expenditure becomes greater under a relatively high level of population aging. It has been found that with the increase of age, the elderly face a higher risk of suffering from various chronic non-communicable diseases (such as cardiovascular diseases, depression, etc.), so they will also suffer from greater mental pressure and negative mood (72). The environmental deterioration, population crowding, increased life pressure, and lifestyle transformation accompanying the process of urbanization have brought many challenges to residents' health. Any urban environment may produce “systematic, socially generated and unfair” health inequalities, which lead to “unhealthy aging” (73). At this time, the aging of the population has increased the probability of being ill. Both disease prevention and treatment have stimulated the growth of demand for medical care, health care, and other services, which has led to a continuous increase in health care expenditures. Therefore, with a higher level of aging, the development of urbanization will promote healthcare expenditure. In the Eastern region, the high level of economic development has brought a low fertility rate, and the rapid development of medical technology and health service has brought about a decrease in the mortality rate. The construction of urbanization also plays a significant role in promoting health care expenditure in this process. The developed economy and low fertility, coupled with the reduction of mortality caused by the rapid development of medical technology and health services, have increased the level of population aging in the Eastern region. The construction of urbanization plays a significant role in promoting health care expenditure in this process.

**Table 3:** Threshold test between urbanization and health care expenditure.

	**Single threshold effect test**			**Double threshold effect test**			
	**Threshold value**	**F-statistics**	***p*-value**	**Threshold value**		**F-statistics**	***p*-value**
East	10.7241	27.01	0.0806	9.2161	14.2507	14.61	0.1278
Center	10.1800	13.66	0.2329	-		-	-
West	7.0054	26.46	0.0150	7.0054	11.6828	11.51	0.2593

**Table 4:** Estimated coefficients of urbanization and health care expenditure.

**Regions**	**Coefficient**	**Estimated value**	***p*-value**
East	${\hat{\alpha }}_{1}$	0.0034***	0.0000
	${\hat{\alpha }}_{2}$	0.0051***	0.0000
Center	${\hat{\alpha }}_{1}$	0.0088	0.1170
	${\hat{\alpha }}_{2}$	0.0105	0.1050
West	${\hat{\alpha }}_{1}$	0.0113*	0.0540
	${\hat{\alpha }}_{2}$	0.0145**	0.0120

***, **, and * indicate significance levels at 1, 5, and 10%, respectively.

The threshold value for population aging is 10.18% in Central China. Below and above this threshold value, the coefficient of urbanization expenditure is 0.0088 and 0.0105, respectively but not significant. Therefore, there is no threshold effect in the Central region. The possible reason is that due to the rapid economic development of cities in some central provinces, livable lifestyles, and more employment opportunities attract a large number of young laborers to flow into the Central region, which continuously pours new vitality into the economic development. Therefore, the role of aging in the impact of urbanization on medical expenditure has not been significant.

In the Western region, the single threshold for aging is 7.00%, which is significant with a 5% significance level. But the double threshold test does not pass the significance test with the *p*-value of 0.2593. When the population aging exceeds the threshold value of 7.00%, the positive effect of urbanization on medical care expenditure will increase from 0.0113 to 0.0145. The threshold value of population aging in the Western region is relatively lower compared with that in the Eastern region. The possible reason is that the Western region's economic and medical development are relatively backward, and people's quality of life is lower than that of the Eastern region. Therefore, when the population aging crosses its threshold (a lower threshold compared with the Eastern region), residents' health care expenditures will increase significantly.

The impact of each control variable in the threshold model is shown in Table 5. Taking the threshold effect into consideration, firstly, the effect of GDP (*lngdp*) on healthcare expenditure is significantly negative at the significance level of 1%, which indicates that economic development can make people's lives healthier and reduce residents' health care expenditure. Secondly, income (*lnincome*) has a significantly positive impact on health care expenditures, and its impact is the greatest among all control variables. Income is the decisive factor that determines people's expenditure level. Higher income can relax consumers' budget constraints and enable consumers to have sufficient money to allocate to medical and health care. Thirdly, the impact of education level on healthcare expenditure is not significant in all regions. According to Grossman's theory of health demand (63), education can have a positive impact on consumer health through production efficiency. However, in China, fierce employment pressure makes people continue to improve their education, and people with higher education often engage in jobs with more tasks and high pressure, which has a negative impact on health. These effects offset each other so that education has no significant impact. Fourth, the effect of medical insurance coverage on health care expenditure is opposite in the Eastern and Central regions. According to China's Sixth National Health Service Survey, the coverage rate of basic medical insurance reached 96.8% in 2021, which means medical insurance has been popularized. But the impact of medical insurance coverage on medical care expenditure is uncertain. On the one hand, medical insurance can relieve some burden of the insured from medical expenditures; on the other hand, a higher level of medical insurance will increase the demand for medical services, which is manifested in more health care spending and reimbursement. Therefore, the direction of the influence from medical insurance on health care expenditure depends on which of these two effects is more dominant. Finally, the number of medical and health institutions can significantly affect the health expenditure in the Central region due to a large number of medical and health institutions in some provinces. According to China's National Health Commission, the number of medical institutions in Central China accounted for 31% in 2019. Among the top 10 provinces in terms of the number of medical institutions, five are from the Central region, namely Henan, Hunan, Shanxi, Jiangxi, and Hubei. More medical resources increase residents' medical care expenditure and contribute to good health, which is consistent with the research of Anand and Barnighausen (74).

**Table 5:** Estimated coefficients of the control variables.

**Regions**	**Variables**	**Coefficient**	**Estimated value**	***p*-value**
East	lngdp	${\hat{\theta }}_{1}$	−0.6225***	0.0010
	lnincome	${\hat{\theta }}_{2}$	0.9953***	0.0000
	edu	${\hat{\theta }}_{3}$	−0.0172	0.6610
	med	${\hat{\theta }}_{4}$	−0.0049**	0.0490
	inst	${\hat{\theta }}_{5}$	−0.0017	0.8270
Center	lngdp	${\hat{\theta }}_{1}$	−0.4481***	0.0000
	lnincome	${\hat{\theta }}_{2}$	0.8883***	0.0000
	edu	${\hat{\theta }}_{3}$	0.0022	0.9460
	med	${\hat{\theta }}_{4}$	0.0279**	0.0290
	inst	${\hat{\theta }}_{5}$	−0.0154**	0.0440
West	lngdp	${\hat{\theta }}_{1}$	−0.3233***	0.0020
	lnincome	${\hat{\theta }}_{2}$	0.8537***	0.0000
	edu	${\hat{\theta }}_{3}$	−0.0421	0.1370
	med	${\hat{\theta }}_{4}$	−0.0007	0.9460
	inst	${\hat{\theta }}_{5}$	−0.0015	0.8990

*** and ** indicate significance levels at 1 and 5%.

## Conclusions

This paper investigates the influence of urbanization on health care expenditure by fixed effect model and uses panel threshold regression model to examine the threshold effect under the background of population aging. The most obvious finding to emerge from this study is that urbanization can significantly contribute to the increase of health care expenditure in Eastern and Central regions. This could be interpreted as a consequence of the developed economy and abundant medical resources. But the impact of urbanization on health care expenditure is not significant in the Western region, which is related to the backward economic development level and low urbanization rate. It should be noted that in the Eastern and Western regions, the impact of urbanization on health care expenditure experiences structural breaks and becomes greater when the level of population aging exceeds the threshold value. This result confirmed that the population aging has stimulated the demand for medical care and services, which has led to an increase in health care expenditures in the process of urbanization with “unhealthy aging”. There are several contributions of this study to this area of research. Firstly, this paper reexamines the impact of urbanization on health care expenditure in China. Under the background of health care reform and the fast development of urbanization, this study has practical and contemporary significance. Secondly, considering the differences among Eastern, Central, and Western regions, this paper further discusses the regional heterogeneity and obtains meaningful conclusions. Thirdly, this paper uses the panel threshold regression model to study whether urbanization has an asymmetric effect on health care expenditure which increases testing power and makes conclusions more accurate.

The empirical results provide some useful implications for policymakers in the face of rapid development of urbanization and increasingly serious population aging. In the urbanization construction, the government should attach great importance to the effect of urbanization on health care expenditure, increase financial support for the construction of medical facilities, and expand the coverage of medical services for residents. The government also needs to increase the coverage of social security, especially to increase the coverage of medical security, to gradually meet the residents' growing medical and healthcare consumption needs. By expanding the risk pool of medical insurance, the risk-sharing among a wider range of insured groups and the enhancement of the mutual assistance of medical insurance can be realized. The government should also focus on the financing of basic medical insurance, reasonably expand financing channels, innovate diversified financing methods, and ensure the sustainable development of medical insurance funds. At the same time, in the context of the deepening of population aging, the government should pay more attention to the development of the medical and health care market for the elderly, especially in the Eastern Region, and build relevant entertainment and health industry to fully meet the medical care needs of the elderly. To deal with the medical and nursing problems caused by population aging, the government can try to take the community as the starting point, make full use of the relative advantages of the community in health services, carry out health examination and medical care services for the elderly residents in the community, and implement elderly medical nursing services.

## Data Availability Statement

The original contributions presented in the study are included in the article/supplementary material, further inquiries can be directed to the corresponding author.

## Author Contributions

QS and ML: data curation, conceptualization, methodology, software, and writing-original draft preparation. RT: data curation and writing-original draft preparation. All authors contributed to the article and approved the submitted version.

## Conflict of Interest

The authors declare that the research was conducted in the absence of any commercial or financial relationships that could be construed as a potential conflict of interest.

## Publisher's Note

All claims expressed in this article are solely those of the authors and do not necessarily represent those of their affiliated organizations, or those of the publisher, the editors and the reviewers. Any product that may be evaluated in this article, or claim that may be made by its manufacturer, is not guaranteed or endorsed by the publisher.

## Footnotes

^1^Eastern region covers 11 provinces: Beijing, Tianjin, Hebei, Liaoning, Shanghai, Jiangsu, Zhejiang, Fujian, Shandong, Guangdong, and Hainan. The Central region includes eight provinces: Shanxi, Jilin, Heilongjiang, Anhui, Jiangxi, Henan, Hubei, and Hunan. The Western region covers 12 provinces, including Chongqing, Sichuan, Guizhou, Yunnan, Inner Mongolia, Tibet, Shaanxi, Gansu, Qinghai, Ningxia, Xinjiang, and Guangxi.
